# Ethnoveterinary practices of Covasna County, Transylvania, Romania

**DOI:** 10.1186/s13002-015-0020-8

**Published:** 2015-05-06

**Authors:** Sámuel Gergely Bartha, Cassandra L Quave, Lajos Balogh, Nóra Papp

**Affiliations:** Department of Pharmacognosy, University of Pécs, Rókus 2, Pécs, 7624 Hungary; Center for the Study of Human Health, Emory University, 550 Asbury Circle, Candler Library 107, Atlanta, GA 30322 USA; Department of Dermatology, Emory University School of Medicine, 1518 Clifton Rd NE, CNR Bldg., Room 5035, Atlanta, GA 30322 USA; Department of Natural History, Savaria Museum, Pf. 14, Szombathely, 9701 Hungary

**Keywords:** Veterinary medicine, Traditional knowledge, Livestock, Székelys, Covasna

## Abstract

**Background:**

Ethnoveterinary medicine is a topic of growing interest among ethnobiologists, and is integral to the agricultural practices of many ethnic groups across the globe. The ethnoveterinary pharmacopoeia is often composed of ingredients available in the local environment, and may include plants, animals and minerals, or combinations thereof, for use in treating various ailments in reared animals. The aim of this study was to survey the current day ethnoveterinary practices of ethnic Hungarian (Székely) settlements situated in the Erdővidék commune (Covasna County, Transylvania, Romania) and to compare them with earlier works on this topic in Romania and other European countries.

**Methods:**

Data concerning ethnoveterinary practices were collected through semi-structured interviews and direct observation in 12 villages from 2010 to 2014. The cited plant species were collected, identified, dried and deposited in a herbarium. The use of other materials (e.g. animals, minerals and other substances) were also documented. Data were compared to earlier reports of ethnoveterinary knowledge in Transylvania and other European countries using various databases.

**Results:**

In total, 26 wild and cultivated plants, 2 animals, and 17 other substances were documented to treat 11 ailments of cattle, horses, pigs, and sheep. The majority of applications were for the treatment of mastitis and skin ailments, while only a few data were reported for the treatment of cataracts, post-partum ailments and parasites. The traditional uses of *Armoracia rusticana, Rumex* spp., powdered sugar and glass were reported in each village. The use of some plant taxa, such as *Allium sativum, Aristolochia clematitis*, and *Euphorbia amygdaloides* was similar to earlier reports from other Transylvanian regions.

**Conclusions:**

Although permanent veterinary and medical services are available in some of the villages, elderly people preferred the use of wild and cultivated plants, animals and other materials in ethnoveterinary medicine. Some traditional ethnoveterinary practices are no longer in use, but rather persist only in the memories of the eldest subset of the population. A decline in the vertical transmission of ethnoveterinary knowledge was evident and loss of practice is likely compounded by market availability of ready-made pharmaceuticals.

## Background

The term “ethnoveterinary” refers to traditional therapeutics prepared by humans for the purposes of maintaining or restoring animal health. The ethnoveterinary pharmacopoeia often contains ingredients sourced from various locations within the environment, and may include plants, animals and minerals. Ethnoveterinary medicine dates back to ancient times and records of this practice can be found in various cultures across the globe. The study of ethnoveterinary medicine through a scientific lens began in the 1970s when it was defined by McCorkle [1], and this subject encompasses theory, taxonomy, diagnosis, practice, resource, and social organization of the health of livestock and pets. Traditional curative and preventive treatments of domesticate and semi-domesticate animals play a significant role in several regions of the world where livestock is a main source of livelihood for rural peoples [2-17].

In Romania, mostly in isolated settlements, several works have been published from the 1960s encompassing data on veterinary health problems of domesticated animals and their management [18-34]. Recently, declines in the transmission and implementation of traditional knowledge have been exacerbated by alteration and degradation of the environment, decreasing numbers of herds, and more expanded availability of officinal medicines and modern pharmaceuticals in several regions of the country. Nevertheless, several ethnic groups preserve the old traditions through home practices and oral transmission of knowledge.

Covasna County, located in the eastern part of Transylvania (situated in central Romania) is inhabited by a population of ethnic Hungarians known as the Székelys. This ethnic group has lived in the Carpathian Basin since the 9th century.

The flora of this area has been studied and published in valuable works [35-39]. Based on these descriptions, the region has a rich flora including relict and endemic species, as well as several medicinal plants used in traditional human and veterinary ethnomedicine [40,41]. In the summary of Rácz and Füzi [41], medicinal plants were listed with local Hungarian, Romanian and scientific names, used part, village and amount of collection (kg/year). Their work highlights the decreasing occurrence of some wild species due to over-harvesting.

Based on our previous ethnobotanical surveys [42,43], the aim of this study was to document and analyze the ethnoveterinary practices of 12 settlements of the Erdővidék commune of Covasna County, Romania, focusing mainly on plant uses, common ailments and homemade therapeutics for livestock (e.g., cattle, horses, sheep and pigs). As no comparative fieldwork has been conducted on veterinary care in Covasna, our collected data were evaluated and compared to records of animal health management practices in Romania and other European countries.

## Methods

### Study area

**Figure 1:**
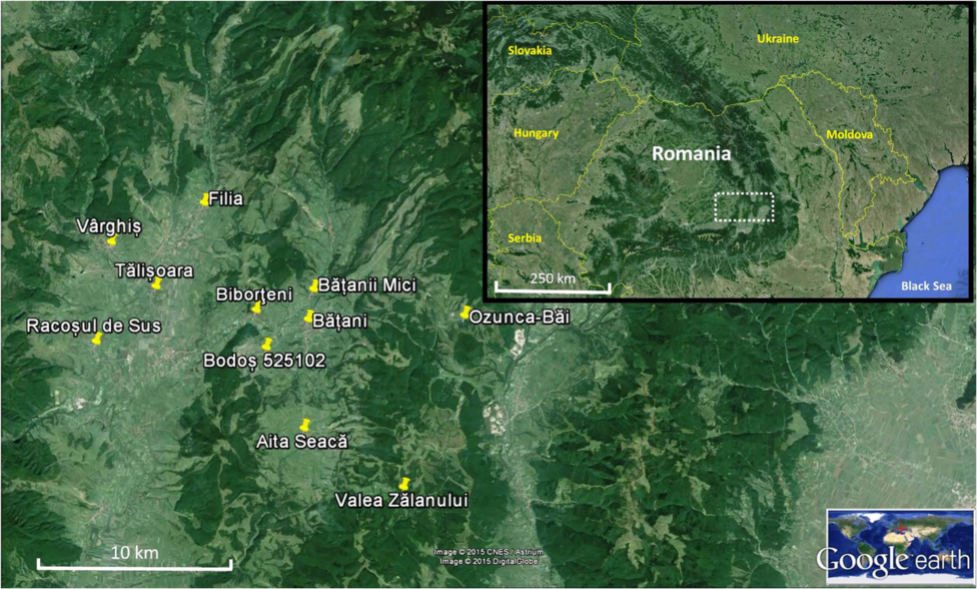
Map of the study sites in Covasna County, Romania [58]. Image adapted from Google Earth (https://earth.google.com/).

Covasna County is located at elevations ranging from 460 to 1,777 m.a.s.l. in eastern Transylvania, situated in central Romania (longitude: 25°28’-26°28’, latitude: 45°32’-46°18’) (Figure 1). The territory encompasses 3,705 km2. This region, which connects to the eastern part of the Carpathian Mountains, has been divided into four large zones: Baraolt Basin, Cîmpul Frumos, Superior Basin of Trei Scaune, Intorsura Buzăului and their surroundings [41]. Erdővidék (“Timberland”) is found in the Baraolt Basin at the north-western part of the county, with a total area of 600 km2. The name “Timberland” comes from the territory being covered with forestland. Average temperatures vary from 2–7°C and the region has a precipitation of 500–1,100 mm per year [44]. The rock-bed consists of vulcanian and sedimental elements. Due to the postvulcanian movement, about 150 mineral springs (“borvízforrás”) were discovered in the region, and are reputed for their medicinal effects. The geological relief of the region is diverse and comprises basins, mountains, valleys, plains and rivers (e.g. Olt, Kormos, Barót and Vargyas). The vegetation is also diverse and comprises beech, birch, hornbeam, oak, and pine forests, and alpine dwarf scrubland at different sea level. In total, the land use area of the county is divided into agriculture (48%), forestry (47.2%) and non-productive surfaces (4.8%) [41].

A 2009 population survey reported the presence of 8,600 ethnic Hungarians (Székely) distributed across 23 villages in Erdővidék [44]. The following settlements were selected in our study: Aita Seacă (in Hungarian: Szárazajta), Băţanii Mari (Nagybacon), Băţanii Mici (Kisbacon), Biborţeni (Bibarcfalva), *Bodoş* (Bodos), Filia (Erdőfüle), Herculian (Magyarhermány), Ozunca-Băi (Uzonkafürdő), Racoşul de Sus (Felsőrákos), Tălişoara (Olasztelek), Valea Zălanului (Zalánpatak), and Vârghiş (Vargyas) (Table 1, Figure 1).

**Table 1:** **Study sites in Covasna County, Romania**

**Study area**	**Latitude**	**Longitude**	**Informants/Inhabitants [** 44 **]**	**Medical service**	**Veterinary practice**	**Pharmacy**	**Local features [** 63 **]**
Aita Seacă	46° 02′ 17″	25° 41′ 23″	7/782	─*	─	─	saline fountain
Băţanii Mari	46° 05′ 22″	25° 41′ 29″	15/1936	+	+	─	mineral springs
Băţanii Mici	46° 06′ 13″	25° 41′ 42″	8/558	─*	─	─	mineral springs
Biborţeni	46° 05′ 37″	25° 39′ 22″	5/775	+	─	+	mineral springs
Bodos	46° 04′ 34″	25° 39′ 36″	9/446	─	─	─	charcoal-burner (“baksa”)
Filia	46° 08′ 38″	25° 37′ 17″	8/1240	+	─	+	iron forge, wooden headbords in the cemetery
Herculian	46° 08′ 02″	25° 42′ 35″	7/1168	+	─	+	mineral springs
Ozunca-Băi	46° 06′ 20″	25° 47′ 20″	3/54	─	─	─	mineral springs, medicinal bath
Racoşul de Sus	46° 04′ 45″	25° 32′ 53″	6/893	─ *	─	─	mineral springs
Tălişoara	46° 06′ 18″	25° 35′ 19″	5/743	─	─	─	mineral springs
Valea Zălanului	46° 00′ 40″	25° 45′ 22″	12/149	─	─	─	mineral springs
Vârghiş	46° 07′ 41″	25° 33′ 25″	14/1647	+	+	+	mineral springs

*Aita Seacă, Băţanii Mici, Racoşul de Sus: no permanent medical service; temporary medical service is available twice per week from from neighbouring communities.

Native people of the county speak Romanian and Hungarian, while in the selected villages the predominant language is Hungarian. The majority of villagers were born in the area and have lived there for most of their lifetime. Many are engaged in traditional agricultural and pastoral activities, working as farmers, ranchers and shepherds. Cattle, goats, horse, sheep and pigs are commonly raised in farms and around the home. They continue to play a key role in the production of dairy products and other traditional foods in the district, as has been the case for centuries. Although some of these villages have access to allopathic medical and veterinary care, as well as access to pharmaceutical drugs (Table 1), most people know of several home treatments for veterinary health problems using materials of various origins.

### Field work and data collection

**Figure 2:**
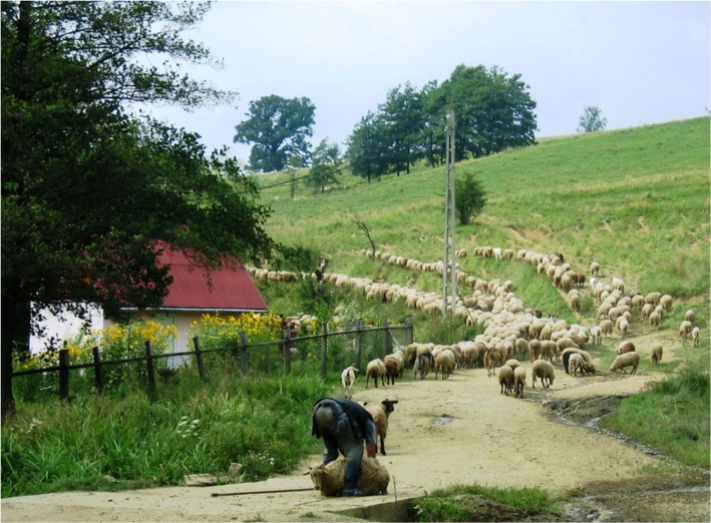
A shepherd works with his livestock in Ozunca-Băi.

Field studies were carried out in the summers of 2010–2014. A total of 99 informants were asked with snow-ball technique in semi-structured interviews in Hungarian. Prior informed consent was obtained prior to conducting interviews and all researchers adhered to the ethical guidelines of the International Society of Ethnobiology [45]. During interviews, details concerning common ailments of domesticated animals, ingredients to traditional therapies (coming from plant, animal, and mineral origin) as well as local healing methods were recorded. Informants were followed into the local agro-ecosytem (e.g. fields, meadows, pastures, ploughlands and road-sides) surrounding villages in order to show and gather the cited wild and cultivated plants (Figure 2). Regarding the cited plant taxa, data concerning the following topics were collected: local name(s), frequency, habitat, time of collection, method of storage, used part, preparation, category and way of use, treated ailments and animals with local name(s), possible beliefs and rituals. Interviews were documented with tape recordings and photos were taken of plants and their habitat as well as the final therapeutic products. Voucher specimens of the cited plants were prepared and deposited at the Department of Pharmacognosy of the University of Pécs. Scientific nomenclature of for botanical taxa followed the systematic work of Tutin et al. [46].

### Data analysis

A search for ethnoveterinary studies in some major databases was conducted and the data collected in this study was compared to earlier documented records in Transylvania and other European countries [3-5,7-17,19-22,25-31,33,34,47-57]. During comparison, similarity and differences of the records were taken into consideration.

## Results and discussion

There are several small private herds widespread in the county. People keep fewer livestock nowadays than in the past, which has resulted in a significant decrease the number of cattle herds. In addition, the prevalence and increased use of agricultural mechanization has led to the reduction in the number of horses necessary for agricultural activities. Nevertheless, domesticated animals continue to play an important role in their everyday life in the region.

Among the 99 informants (63 women and 36 men) aged between 27 and 99 years, only 75 villagers reported that they currently raise cattle, horses, sheep or pigs and use ethnoveterinary treatments. While the other 24 informants do not currently rear livestock, they did in the past and where able to provide information regarding past ethnoveterinary practices used during their lifetime. Declines in the transmission of traditional ethnoveterinary knowledge are apparent, and is likely linked to emigration trends among young people seeking employment in larger cities and even foreign countries. In addition, many informants commented on a sense of greater faith in modern veterinary medicines prescribed by veterinarians than their own home remedies.

Altogether, 11 ailments reported to be most frequently treated via ethnoveterinary means (Tables 2,3,4). Among the cited ailments, “hotness” (stomach disorders), inflamed udder (mastitis), respiratory diseases (roaring = “kehesség”, infectious cold, cough, pneumonia), rumination disorders, wounds and skin injuries, diarrhea, and cataracts were listed, and local methods were also used in post-partum therapies and as anthelmintic, diuretic, antiparasitic, repellent and insecticide drugs. The majority of ethnoveterinary therapeutics were observed to treat the ailments of cattle (48 records), while 26 remedies were mentioned for horses, 15 for pigs, and 5 for sheep.

**Table 2:** **Plant taxa used in ethnoveterinary medicine of the selected villages**

**Used plants and voucher code**	**Family**	**Local name**	**Medicinal use**	**Status** ^*^	**Number of citations**
*Achillea millefolium* L. (EV-03)	Asteraceae	*cickafarok, cickafarkkóró, pulykafű, egérfarkú virág, egérfarkúfű, egérfarkúfűvirág*	herb as a tea for rumination [cattle]	W	3
*Allium sativum* L. (EV-22)	Amaryllidaceae	fokhagyma	bulb for anthelmintics [pig]	C	76
*Aristolochia clematitis* L. (EV-23)	Aristolochiaceae	farkasalma	leaf for wounds and skin injuries [cattle, horse, pig, sheep]	W	54
*Armoracia rusticana* G. Gaertn., B. Mey. & Schreb. (EV-11)	Brassicaceae	torma	root for respiratory disorders [horse]	C	71
*Artemisia absinthium* L. (EV-13)	Asteraceae	*üröm,* fehér üröm	herb for stomach heat, inflammation, and ache [cattle]	W	4
			for diarrhea [cattle, horse]		5
*Calendula officinalis* L. (EV-12)	Asteraceae	*sárgavirág*	flower as a cream for inflamed udder and mastitis [cattle]	C	4
*Cucurbita pepo* L. (EV-21)	Cucurbitaceae	tök	ground seed and seed oil for rumination [cattle]	C	3
			for anthelmintics [pig]		2
*Daucus carota* L. subsp. *sativus* Hoffm. (EV-04)	Apiaceae	*murok*	root for anthelmintics [horse]	C	4
*Eryngium planum* L. (EV-17)	Apiaceae	*kék tilinkó, szamárcsipke, bojtorján*	herb for inflamed udder and mastitis [cattle, horse]	W	7
*Euphorbia amygdaloides* L. (EV-01)	Euphorbiaceae	*árió*	herb for wounds and skin injuries [horse, pig]	W	6
*Gentiana asclepiadea* L. (EV-15)	Gentianaceae	*gyertyagyökerű, gyertyagyükerű, sárgagyökér*	root as a tea for stomach heat, inflammation, and ache [cattle]	W	4
*Juglans regia* L. (EV-16)	Juglandaceae	dió	leaf against flies as a rub [horse]	C	47
*Juniperus communis* L. (EV-14)	Cupressaceae	*borsika*	pseudofruit for respiratory disorders [horse]	W	6
*Matricaria chamomilla* L. (EV-10)	Asteraceae	*kamillavirág*	flower as a tea and wash for inflamed udder and mastitis [cattle]	W	11
*Petroselinum crispum* (Mill.) Fuss (EV-09)	Apiaceae	*ződ peterzselyem*	leaf for rumination and after delivery [cattle]	C	9
			for diuretics [horse]		5
*Plantago lanceolata* L. (EV-25)	Plantaginaceae	*kígyónyelvűfű hegyesútilapi, kígyónyelvű útifű, keskeny útifű lándzsás/keskeny útilapi*	leaf for stomach heat, inflammation, and ache [cattle]	W	12
*Polygonum minus* Huds. (EV-02)	Polygonaceae	*árió, veresszárúfű*	herb as a washing for wounds and skin injuries [cattle, horse]	W	11
*Potentilla anserina* L. (EV-05)	Rosaceae	*pipefű, lúdlábfű, lúdfű*	leaf for diarrhea [cattle, horse, pig, sheep]	W	71
*Quercus petraea* (Matt.) Liebl. (EV-19)	Fagaceae	*cserefa, cseremakk, cserháncs*	nut for anthelmintics [pig, cattle]	W	4
			young bark as a tea for diarrhea [pig]		5
*Quercus robur* L. (EV-20)	Fagaceae	*cserefa, cseremakk, cserháncs*	nut for anthelmintics [pig, cattle]	W	4
			young bark as a tea for diarrhea [pig]		5
*Rumex acetosella* L. (EV-07)	Polygonaceae	*lósósdi, lósóska*	fruit for diarrhea [cattle, horse, pig, sheep]	W	75
*Rumex obtusifolius* L. (EV-06)	Polygonaceae	*lósósdi, lósóska*	fruit for diarrhea [cattle, horse, pig, sheep]	W	75
*Salix alba* L. (EV-24)	Salicaceae	fűzfa	leaf as fodder for stomach heat, ache, inflammation, and rumination, leafy branches and bark for rumination [cattle]	W	24
*Secale cereale* L. (EV-26)	Poaceae	rozs	fruit for anthelmintics [horse]	C	4
*Symphytum officinale* L. (EV-18)	Boraginaceae	*fekete nadály, nadály, forrasztófű*	root with bran for rumination [cattle]	W	5
*Veratrum album* L. (EV-08)	Melanthiaceae	*ászpa,* (fehér)zászpa	ground root as a rub against lice [horse]	W	5

^*^ *Status:* W = growing in wild habitat; C = cultivated in gardens.

**Table 3:** **Animals and other materials used in ethnoveterinary medicine of the study area**

**Animals**	**Medicinal use**	**Number of citations**
*Menyet* (*Mustela nivalis* L.)	skin as an embrocation by itself or soaked in milk for mastitis [cattle]	29
Tetű (lices)	put into the urethra as a diuretic [horse]	3
**Minerals and other substances**		**Number of citations**
Bread	for rumination [cattle]	11
Clay	by itself or with salt as an embrocation for mastitis [cattle]	9
Cobweb	as an embrocation for skin injuries [horse]	3
“hótszén” (hot embers quenched in water)	for diarrhea [pig]	4
Glass (powdered)	blown into the eyes for cataract [cattle]	63
“szénamurha” (hay)	by itself or poured with urine for respiratory diseases [horse]	31
Injection	for respiratory diseases [horse]	3
Milk	for stomach heat, inflammation, ache [cattle], with *Allium sativum* as an anthelmintic [pig]	22
Oil	for stomach heat, inflammation, ache, rumination [cattle]	25
Salt	with water and vinegar as a wash or embrocation for mastitis [cattle]	7
Sugar (powdered)	blown into the eyes for cataract [cattle]	61
Turpentine	for respiratory diseases [horse]	2
Toast	for rumination [cattle]	9
Urine (human)	poured onto “szénamurha” for respiratory diseases [horse]	5
Vinegar	with water and salt as a wash or embrocation for mastitis [cattle]	18
Water	with vinegar and salt as a wash or embrocation for mastitis [cattle]	19
	poured beside the animals as a diuretic [horse]	7
	put into the shed to vaporize and induce urination [sheep]	6
Whey powder	anthelmintics [pig]	3

**Table 4:** **Ethnoveterinary practices in the study area compared with earlier records in Romania and some European countries**

**Diseases treated in ethnoveterinary practice**	**Ethnomedicinal treatments in the studied villages** ^*^		**Earlier reported data in Romania**		**Data in other countries**	
	Used ingredients	Parts used and preparation	Used ingredients	Parts used and preparation	Used ingredients	Parts used and preparation
“hotness” (stomach heat, inflammation, ache)	*Artemisia absinthium*	herb^1,4^ [cattle]	*Centaurium erythraea* Rafn. (Gentianaceae)	herb as tea [pig] [47]	*Achillea millefolium*	flowers as infusion [53]
	*Plantago lanceolata*	leaf with the herb of *A. absinthium* ^1^ [cattle]	*Levisticum officinale* W.D.J. Koch (Apiaceae)	herb with rancid pork fat and bitter salt [48]	*Matricaria chamomilla*	flowers as infusion [calves] [51]
	*Gentiana asclepiadea* (Figure 3)	root with *P. lanceolata* as a tea^12^ [cattle]	*Peucedanum oreoselinum* Moench (Apiaceae)	herb as fodder [48]	*Potentilla erecta* (L.) Raeusch. (Rosaceae)	root as a tea [51]
	*Salix alba*	leaf as fodder^12^ [cattle]	*Rumex crispus* L. (Polygonaceae)	seed soaked in brandy for digestive problems [48]	milk	[7]
	milk	[cattle]^5^				
	oil	[cattle]^5,9^				
inflamed udder, mastitis	*Calendula officinalis*	flower as a cream^2^ [cattle, horse]	*Brassica oleracea*	sour leaf sap with human urine and dung of horse as a cream [47]	*Brassica oleracea*	roasted leaf as an embrocation [14]
	*Eryngium planum*	herb as a tea^2^ or washing^12^ [cattle, horse]	*Calendula officinalis*	flower as a cream [47]	*Calamintha nepeta* (L.) Savi (Lamiaceae)	leaf as a wash [15]
	*Matricaria chamomilla*	flower as a tea and wash^12^ [cattle]	*Digitalis grandiflora* Mill. (Plantaginaceae)	herb [26]	*Malus sylvestris* (L.) Mill. (Rosaceae)	cider vinegar of fruit with grain as fodder [11]
	*Mustela nivalis*	skin as an embrocation by itself^2,6,9,11^ or soaked in milk^7^ [cattle]	*Malus sylvestris*	vinegar of fruit on slate as impregnant, as an embrocation [cattle] [28,47]	*Malva sylvestris*	fresh leaf boiled and placed into sack using as a warm compress [15]
	cold water	as a wash^1,2,6,12^; with vinegar^2,3^ and salt^7^ as a wash^2,7^ or embrocation^3^ [cattle]	*Mustela nivalis*	as a rubber [49]	*Olea europaea* L. var. *europaea* (Oleaceae)	fruit as an ointment [16]
	clay	by itself or with salt as an embrocation^7^ [cattle]	*Papaver somniferum* L. (Papaveraceae)	seed as fodder for “reszfug” (= mastitis) [cattle, sheep] [26]	*Sambucus nigra* L. (Adoxaceae)	flower in fumigation [cattle] [16]
			*Scrophularia nodosa* L., *részfugburján* (Scrophulariaceae)	as a wash [26], mixed and cooked with salt and flour of *Zea mays* [19] dried and mixed into the flour as fodder [20]	*Thymus vulgaris* L. (Lamiaceae)	decoction of flowering stem as a wash [cattle, dog, sheep] [8]
			*Picea abies* (L.) H. Karst. (Pinaceae)	resin by itself, or with sour cream or tallow as an embrocation [Papp, unpublished data, Uz-valley]	*Zea mays* L. (Poaceae)	seed as a decoction in water and/or milk as a washing [cattle] [8]
			flour	with salt [26]		
			water	with vinegar and salt as a washing and an embrocation [34]		
			yellow mud	smeared onto the udder [cattle] [28]		
respiratory diseases: roaring (“kehesség”), cold, cough, pneumonia	*Armoracia rusticana*	root as fodder^1–12^ [horse]	*Allium cepa* L. (Amaryllidaceae)	3 slices of the bulb grated and soaked in brandy, and mixed with saltpetre [29]	*Allium cepa*	bulb [16]
			*Armoracia rusticana*	root by itself [34,47], or with *Avena sativa* and urine for “száraz kehe” (dry cough) of horse [28,29], or in boiled milk with one spoon of honey, tallow, yeast, 7 slices of *Allium sativum*, and 9 fruits of *Pimenta dioica* (L.) Merr., Myrtaceae and *Syzygium aromaticum* (L.) Merr. & L.M. Perry, Myrtaceae and *Piper nigrum* L., Pipeaceae [horse] [29], or with “büdöskővirág” (sulphur powder) [34]	*Atropa belladonna* L. (Solanaceae)	leaf [horse, dog] [8]
	*Juniperus communis*	pseudofruit as fodder^7^ [horse]	*Avena sativa* L., Poaceae	warmed by itself [28], or with urine, turpentine or “büdöskővirág” put into a sac and pull onto the head of horse as a steaming	*Avena sativa*	aerial part [horse] [10]
	injection	[horse] ^1^	*Brassica oleracea*	leaf sap dropped into the nostrils with dried and ground rat snake [horse] [19]	*Eucalyptus globulus* Labill. (Myrtaceae)	leaf [16]
	“szénamurha”	by itself^1,5,12^ or poured with urine^7^ [horse]	*Datura stramonium* L. (Solanaceae)	leaf [30]	*Helleborus bocconei* Ten. (Ranunculaceae)	petiole inserted into the ear or the neck for bronchitis [cattle] [13]
	turpentine	[horse]^1^	*Helleborus purpurascens* Waldst. & Kit. (Ranunculaceae)	leaf soaked in whey [19], or pulled into the breast [horse, cow], and into the ears [pig] [20,48,50] for dry and purulent cough [34]	*Helleborus foetidus* L. (Ranunculaceae) [cattle][12]	leaf inserted into the ears for bronchitis and pneumonia [cattle] [12]
			*Hordeum vulgare* L., *H. vulgare* convar. *vulgare* (Poaceae)	steaming with warmed seed [27,31], flour with honey and water [29]	*Juniperus phoenicea* L. (Cupressaceae)	leaf [cattle, sheep, dog, horse] [8]
			*Juniperus communis*	as a tea [horse] [25]	*Mercurialis annua* L. (Euphorbiaceae)	root as a tea [dog] [8]
			*Levisticum officinale* W.D.J. Koch (Apiaceae)	aerial part as a tea [21,48]	*Origanum heracleoticum* L. (Lamiaceae)	aerial part [4]
			*Matricaria chamomilla*	flower as a tea [pig] [47]	Sugar	on hot coal as a fumigant [4]
			*Malus sylvestris*	vinegar of the fruit as an embrocation [47]	tin	melted and inserted into the nose [horse] [10]
			*Pulmonaria officinalis* L. (Boraginaceae)	flower for pneumonia [pig] [19,20]		
			*Secale cereale* L. (Poaceae)	flour for “fojókehe” for steaming [28,29]		
			*Triticum aestivum* L. (Poaceae)	bran by itself [29]		
			bear, goose and pork fat	for “csikókehe, fojtókehe” [horse] [29]		
			salt	sprinkled onto the nose [33]		
			sulphur powder	for “csikókehe, fojtókehe” [horse] [29]		
			venesection	[33]		
rumination	*Achillea millefolium*	herb as a tea [cattle]^12^	*Allium sativum*	bulb with bread [28,29], or with wine and egg [33]	*Achillea millefolium*	aerial part [cattle] [54,56]
	*Cucurbita pepo*	ground seed and seed oil [cattle]^2^	*Angelica sylvestris* L. (Apiaceae)	leaf [21]	*Artemisia absinthium*	aerial part as a tea [10]
	*Petroselinum crispum*	leaf [cattle]^2^	*Armoracia rusticana*	roasted fruit [29]	*Pimpinella anisum* L. (Apiaceae)	fruit as an elixir [10]
	*Salix alba*	leaf, leafy branches and bark [cattle]^3^	*Artemisia dracunculus*	herb as a tea [29]	*Ruta chalepensis* L. (Rutaceae)	aerial part as a tea [16]
	*Symphytum officinale* L.	root as fodder with bran [cattle]^12^	*Avena sativa*	roasted fruit [29]	*Salix purpurea* L., (Salicaceae)	branches [10]
	bread	[cattle]^3,4^	*Beta vulgaris* L. convar. *crassa* (Amaranthaceae)	grated root [21]	*Tanacetum parthenium* (L.) Schultz Bip. (Asteraceae)	aerial part as a tea [16]
	Toast	[cattle] ^7^	*Cannabis sativa* L. (Cannabaceae)	seed in oil [29]	Beer	[cattle] [10]
	Oil	[cattle] ^7^	*Carum carvi* L. (Apiaceae)	fruit [21]	Buttermilk	[cattle] [10]
			*Cucurbita maxima* Duchesne (Cucurbitaceae)	seed with bran [22]	soda (sodium bicarbonate)	[cattle] [10]
			*Cucurbita pepo*	ground seed with milk [28,30]	whey	[cattle] [10]
			*Daucus carota* ssp. *sativus* Hoffm.	root [21,47]		
			*Euonymus europaeus* L. (Celastraceae)	fruit [21]		
			*Equisetum sylvaticum* L. (Equisetaceae)	herb [27,31]		
			*Fragaria vesca* L. (Rosaceae)	fruit [30] or root as a tea [28]		
			*Helianthus annuus* L. (Asteraceae)	pressed seed coat [21,47]		
			*Iris germanica* L. (Iridaceae)	root [20]		
			*Juniperus communis*	pseudofruit with milk, roasted on bread [28], or woth the leaf of *Salix alba*, rusty fat, oil and bulb of *Allium cepa* [30,47]		
			*Levisticum officinale* W.D.J. Koch (Apiaceae)	herb [21]		
			*Linum usitatissimum* L. (Linaceae)	seed as a tea [21,24,31,34]		
			*L. usitatissimum* convar. *Transitorium* (Linaceae)	ground seed with the seed of *Helianthus annuus* [29]		
			*Malus domestica* Borkh.	vinegar with yeast and *Artemisia dracunculus* [29]		
			*Matricaria chamomilla*	flower as a tea [20]		
			*Petroselinum crispum*	leaf with bran and oil [22]		
			*Peucedanum oreoselinum* Moench (Apiaceae)	herb [21]		
			*Prunus domestica* L. ssp*. Rotunda* (Rosaceae)	leafy branches [28]		
			*Raphanus sativus* L. cv. *niger* f. s*ubglobosa* (Brassicaceae)	tuber [21,24,31,34], or with cooking soda [22]		
			*Rumex stenophyllus* Ledeb.(Polygonaceae)	herb [31]		
			*Rubus idaeus* L. convar. *hortensis* provar. *inermis* (Rosaceae)	fruit as a syrup [29]		
			*Sambucus nigra*	lower layer of the bark [30]		
			*Salix alba*	leafy branches [28,30]		
			*Salix* spp.	leafy branches [31]		
			*Satureja hortensis* L. (Lamiaceae)	herb [28]		
			*Sisymbrium strictissimum* L. (Brassicaceae)	root [28]		
			*Triticum aestivum*	fruit [29]		
			*Zea mays*	stem [29]		
			copper sulphate	[34]		
			white wine	with egg [sheep] [33]		
wounds, skin injuries	*Aristolochia clematitis*	leaf as an embrocation [cattle, horse, pig, sheep]^1,2,4,6–11^	*Achillea millefolium*	herb cut and mixed with rancid fat [25]	*Acer pseudoplatanus* L. (Sapindaceae)	decoction of the bark as a wash [9]
	*Eryngium planum*	herb as a tea [cattle, horse]^2^	*Aristolochia clematitis*	decoction of the stem and leaf as a wash, or the leaf as an embrocation [21,28,30]	*Althaea officinalis* L. (Malvaceae)	root [9]
	*Euphorbia amygdaloides*	herb as a tea [horse, pig]^12^	*Betula pendula* Roth. (Betulaceae)	leaf for bruised skin (“pecsendzsia, pokolszökés”) as a tea [50]	*Agave americana* L. (Asparagaceae)	leaf [9]
	*Polygonum minus*	as a washing [cattle, horse]^10^	*Chelidonium majus* L. (Papaveraceae)	leaf sap [47]	*Artemisia absinthium*	aerial part with honey [horse] [10]
	cobweb	as an embrocation [horse]^5^	*Crataegus monogyna* Jacq. (Rosaceae)	fruit or leafy branches as a decoction [ox] [25]	*Bovista dermoxantha* Pers. (Lycoperdaceae)	old fruiting body [horse] [10]
			*Daphne mezereum* L. (Thymelaeaceae)	bark [sheep] [20], flower as an infusion [horse] [Papp, unpublished data, Uz-valley]	*Cardopatum corymbosum* (L.) Pers. (Asteraceae)	leaf [sheep,cattle, dog] [8]
			*Euphorbia amygdaloides* (Euphorbiaceae)	ground herb ss an embrocation [26], or as a wash ([28,30,47] Papp, unpublished data, Uz-valley)	*Carpinus orientalis* Mill. (Betulaceaeae)	bark as a decoction [3]
			*Euphorbia cyparissias* (Euphorbiaceae)	herb as a wash [28,30]	*Centaurea alba* L. ssp. *tartesiana* Talavera (Asteraceae)	leaf [horse] [8]
			*Euphorbia palustris* L. (Euphorbiaceae)	herb as a wash [28,30]	*Daphne gnidium* L. (Thymelaeaceae)	stem as a liniment [9]
			*Polygonum lapathifolium* L. (Polygonaceae)	leaf as an embrocation [26]	*Ecballium elaterium* (L.) A. Rich. (Cucurbitaceae)	fruit [4]
			*Symphytum officinale*	root [20]	*Euphorbia hirsuta* L. (Euphorbiaceae)	[sheep, cattle, horse, dog] [51]
			*Veronica beccabunga* L. (Plantaginaceae)	herb as a wash [19]	*Gentiana lutea* L. (Gentianaceae)	root as a bath [9]
			cobweb	[19]	*Geranium rotundifolium* L. (Geraniaceae)	aerial part [9]
			hot fat	[19]	*Hypericum perforatum*	aerial part [3,8,51]
			lime	[19]	*Juniperus oxycedrus* L. (Cupressaceae)	pseudofruit as an ointment [9]
			urine	[19]	*Lilium pyrenaicum* Gouan (Liliaceae)	bulb as a liniment and a poultice [9]
					*Malva neglecta* Wallr. (Malvaceae)	aerial part [3], leaf [51]
					*Marrubium vulgare* L. (Lamiaceae)	aerial part [sheep, cattle] [8]
					*Nicotiana tabacum* L. (Solanaceae)	leaf [sheep] [9]
					*Peucedanum ostruthium* (L.) W.D.J.Koch (Apiaceae)	root as an ointment and bath [51]
					*Prunus domestica*	fruit by itself [8], or in fermented and distilled form [7]
					*Pulicaria odora* Rchb. (Asteraceae)	flowering top in alcohol or as a decoction [cattle, sheep, dog, horse] [8]
					*Quercus ilex* L. ssp. *ilex*, *Q. petraea* (Matt.) Liebl. (Fagaceae)	bark as a bath [9]
					*Ruta chalepensis*	aerial part as an embrocation and liniment [9]
					*Sambucus nigra*	leaf as a decoction [14]
					*Symphytum officinale*	root as an ointment and bath [51]
					*Valeriana officinalis* L. (Caprifoliaceae)	root or leaf in mules [14]
					fat	fox fat for pimples [horse], hen fat for sores [oxen] [2]
					Sulphur	in water for burns [10]
					Cobweb	[7]
anthelmintics	*Allium sativum*	bulb of by itself^1,3–6,8–12^ or soaked in milk [pig]^2,12^	*Allium sativum*	bulb by itself [28,30,34] or in milk [34]	*Allium sativum*	bulb mixed with oil [dog] [8,14], or in water [3]
	*Cucurbita pepo*	seed [pig]^3,12^	*Armoracia rusticana*	root with the pseudofruit of *Juniperus communis* [30]	*Artemisia absinthum*	leaf as a decoction [dog] [8]
	*Daucus carota* ssp. *sativus*	root [horse]^7^	*Avena sativa*	roated fruit [28]	*Artemisia herba-alba* Asso (Asteraceae)	aerial part [sheep] [8]
	*Quercus petraea, Q. robur*	nut [pig, cattle]^3^	*Cannabis sativa*	seed with lime-water, or with Allium cepa, goose fat and milk [28]	*Chelidonium majus* L. (Papaveraceae)	leaf in water [3]
	*Secale cereale*	fruit as fodder [horse]^7^	*Cucurbita pepo*	seed with the seed of *Ricinus communis* L., (Euphorbiaceae)	*Chenopodium ambrosioides* L. (Amaranthaceae)	aerial part [3,16]
	whey powder	[pig]^2^	*Dryopteris filix-mas* (L.) Schott, (Dryopteridaceae)	dried rhizome [poultry] [29,41]	*Daphne gnidium*	bark [swine, cattle, sheep, dog, horse] [8]
			*Hordeum vulgare*	roasted seed [27,29,31]	*Hypericum maculatum* Crantz (Hypericaceae)	aerial part as a tea [7]
			*Phaseolus vulgaris* L. Fabaceae	fruit as a decoction without salt [29]	*Mentha suaveolens* Ehrh.(Lamiaceae)	aerial part as a tea [16]
			*Quercus petraea, Q. robur*	ground nut or bark as a tea [28]	*Ruta chalepensis*	aerial part [3,16]
			*Sambucus nigra*	bar kin milk [25,30]	*Scabiosa columbaria* L. (Caprifoliaceae)	aerial part as a tea [16]
			*Secale cereale*	fruit or flour [30]	*Simethis mattiazzi* (Vand.) Sacc. (Xanthorrhoeaceae)	root as a decoction [3]
			*Triticum aestivum*	bran with butter, fat, salt and soap as a decoction [29]	*Teucrium scorodonia* L. (Lamiaceae)	aerial part [3,16]
			bran	with ash [33]		
			dove dung	dried dung mixed into the fodder [34]		
			petroleum	[33]		
diarrhea	*Artemisia absinthium*	herb as a tea [cattle, horse]^3^	*Achillea collina* (Becker ex Rchb. f.) Heimerl, *A. millefolium* (Asteraceae)	aerial part as a tea [22,26]	*Achillea millefolium*	aerial part [calf] [10]
	*Potentilla anserina*	leaf as a tea [cattle, horse, pig, sheep]^1,2,4–6,9–12^	*Alchemilla vulgaris* L. (Rosaceae)	aerial part as a tea [41]	*Achillea ptarmica* L. ssp. *pyrenaica* (Sibth. ex Godr.)	flower with the flower of *Sambucus nigra* as a tea [16]
	*Rumex acetosella, R. obtusifolius*, *Rumex* spp.	fruit as a tea [cattle, horse, pig, sheep]^1–12^	*Alnus glutinosa* (L.) Gaertn. (Betulaceae)	bark as a decoction [48]	*Agrimonia eupatoria* L. (Rosaceae)	aerial part as a tea [16]
	*Quercus petraea, Q. robur*	bark as tea [pig]^3^	*Artemisia absinthium*	aerial part as a tea [28]	*Brassica oleracea* ssp. *oleracea*	aerial part [16]
	“hótszén”	as fodder [pig]^2^	*Aesculus hippocastanum* L. (Sapindaceae)	seed [cattle, pig] [28,29,34]	*Ceratonia siliqua* L. (Fabaceae)	grain [51]
			*Chelidonium majus*	aerial part as a tea [26]	*Chelidonium majus*	leaf as a tea [3]
			*Equisetum arvense* L. (Equisetaceae)	aerial part as a tea [48]	*Citrus limon* (L.) Burm. (Rutaceae)	epicarp of the fruit with the seed of *Oryza sativa* L. (Poaceae) as a soup [51]
			*Fagus sylvatica* L. (Fagaceae)	bark as a decoction [cattle] [34,47]	*Eriobotrya japonica* (Thunb.) Lindl. (Rosaceae)	leaf as a tea [16]
			*Hordeum vulgare*	roasted seed [pig, horse] [22,33], or with salty flour with the seed of *Secale cereale* [29]	*Daphne gnidium*	stem [16]
			*Juniperus communis*	pseudofruit as a tea [cattle] [25]	*Foeniculum vulgare* Miller (Apiaceae)	aerial part [16]
			*Quercus cerris* L.	ground bark as a tea [47]	*Hypericum maculatum*	aerial part as a tea [7]
			*Quercus petraea*	ground bark in fodder [22]	*Lythrum salicaria* L. (Lythraceae)	aerial part [16]
			*Rumex acetosa* L., *R. confertus* Willd., *R. crispus*	seed as a decoction [19,20,22,28,30,48]	*Phlomis purpurea* L. (Lamiaceae)	aerial part [sheep, horse, dog] [8]
			*Rumex acetosella*	seed as a decoction [47]	*Quercus rubra* L. (Fagaceae)	branch [rabbit] [8]
			*Rumex patientia* L., *R. stenophyllus* Ledeb.	[cattle, horse, pig] [31]	*Polygonum aviculare* L. (Polygonaceae)	aerial part as a tea [8]
			*Ruta graveolens* L. (Rutaceae)	aerial part as a tea [cattle] [29]	*Potentilla reptans* L. (Rosaceae)	aerial part as a tea [rabbit] [8]
			*Sambucus racemosa* L. (Adoxaceae)	fruit as a tea [48]	*Rosmarinus officinalis* L.	aerial part as a tea [16]
			*Sisymbrium strictissimum* L. (Brassicaceae)	root as a decoction [cattle] [30]	*Rumex acetosella*	aerial part [cattle] [5]
			*Solanum tuberosum* L. (Solanaceae)	tuber with the leaf of *Robinia pseudoacacia* L. (Fabacaeae) [48]	*Rumex* sp.	boiled seeds in water [pig] [64]
			*Sorbus domestica* L. (Rosaceae)	bark as a decoction [20]	*Thymus serpyllum* L. ssp. *nervosus* (Willk.) Nyman (Lamiaceae)	aerial part as a tea [16]
			*Triticum aestivum*	bran by itself [calf] [21]	*Vaccinium myrtillus* L. (Ericaceae)	raw or dried fruit [51]
			ash	[horse] [33]	*Verbascum sinuatum* L. (Scrophulariaceae)	flower as a tea [16]
			salt	[sheep] [48]		
			vinegar	[sheep] [48]		
diuretics	*Petroselinum crispum*	leaf [horse]^3^	*Allium cepa*	bulb as a decoction [cattle, horse] [34], or into the urethra [horse] [19-21]	*Daphne laureola* L.	aerial part [3]
	ammonia	to smell in the stable [horse]^5,6^	*Capsicum annuum* convar. *longum*, (Solanaceae)	fruit [29]	*Herniaria hirsuta* L. ssp. *cinerea* (DC.) Coutinho (Caryophyllaceae)	aerial part [16]
	lice	put into the urethra [horse]^5^	*Fragaria vesca* L. (Rosaceae)	root as a tea [20]	*Rorippa nasturtium-aquaticum* (L.) Hayek, (Brassicaceae)	as a decoction [pig] [8]
	water	poured beside the animals [horse]^7^, or put into the shed to vaporize and induce urination [sheep]^7^; animals guided to the edge of rivers to hear the sound of water [horse]^5^	*Narcissus stellaris* Haw. (Amaryllidaceae)	flower [cattle] [30]	*Simethis mattiazzi* (Vand.) Sacc. (Xanthorrhoeaceae)	root [3]
			*Petroselinum crispum*	leaf or root with saltpeter as a decoction [cattle, horse] [19-21,34]	*Zea mays*	stigma as a decoction [3]
			*Peucedanum* sp. (Apiaceae)	boiled herb as fodder [sheep] [48]		
			*Polygonum bistorta* L. (Polygonacaeae)	rhizome [26]		
			*Sambucus nigra*	flower as a decoction [cattle] [33]		
			*Urtica dioica* L. (Urticaceae)	root as a tea [19]		
cataract	powdered sugar	blown into the eyes [cattle]^1–12^	*Anagallis arvensis* L. ssp. *phoenicea* Vollmann (Primulaceae)	dried and ground petals mixed with powdered sugar [22]		
	powdered glass	blown into the eyes [cattle]^1–12^	*Capsicum annuum*	dried and ground pungent fruit [cattle] [21,48]		
			*Malus sylvestris*	vinegar of the fruit as an embrocation [47]		
			*Matricaria chamomilla*	tea of the flower as a wash [22]		
			*Nicotiana tabacum*	ground leaf spit with saliva into the eyes [47]		
			*Tilia cordata* Mill. (Malvaceae)	yellow part under the bark with milk as an embrocation [47]		
			“szentgyörgybéka” (*Bombina variegata* L., Bombinatoridae)	put into the eyes [19,49]		
			ash	blown into the eyes [cattle] [49]		
			salt	blown into the eyes [horse] [19,33]		
			sugar	blown into the eyes [cattle] [19,33,49]		
			powdered glass	blown into the eyes [horse] [19,33]		
			powdered porcelain	blown into the eyes [33]		
after delivery	*Petroselinum crispum*	leaf to promote expulsion of the placenta as fodder [cattle]^3^	*Hordeum vulgare* convar. *vulgare*	roasted seed as fodder [cattle] [29]		
antiparasitic, repellent, and insecticide effect; for scab	*Juglans regia*	leaf against flies as a rub [horse]^6^	*Aconitum moldavicum* Hacq. (Ranunculaceae)	root against lice and ticks [26]	*Capsicum annuum*	fruit in oil [16]
	*Veratrum album*	ground root as a rub against lice, flies and mosquitos [horse]^6^	*Artemisia absinthium*	aerial part strewed against lice, spray with lime in the chicken pen [33]	*Cestrum parqui* L 'Hér. (Solanceae)	herb [52]
			*Ballota nigra* L. (Lamiaceae)	aerial part put under hen against lice [31]	*Juglans regia*	leaf against flies as a decoction [horse] [16]
			*Brassica oleracea, B. oleracea* var. *capitata*	sour sap of the leaf against scab [sheep] [19,20], salty leaf sap against ox warble fly [28] and lice [29]	*Laurus nobilis* L. (Lauraceae)	fruits in olive oil [52]
			*Cucurbita pepo*	runner against flies as a rub [29]	*Matricaria chamomilla*	flowering top against fleas [cattle, sheep, dog] [8]
			*Dryopteris filix-mas*	leaf as bed of straw [pig] [25]	*Olea europaea* var. *europaea*	seed oil as a repellent poultice [16]
			*Euonymus europaeus* L. (Celastraceae)	dried and ground fruit against lice [cattle, hen, pig] [28,29]	*Pteridium aquilinum* (L.) Kuhn (Dennstaedtiaceae)	leaf against fleas [16]
			*Helianthus annuus*	seed oil smeared onto the animals against lice [cattle, hen, pig] [28,29]	*Ruta chalepensis*	aerial part against flies as a rub [horse] [8], and against fleas [16]
			*Helleborus purpurascens*	root as a decoction against scab [30]	*Sonchus oleraceus*	aerial part in oil and as a poultice against flies [horse, cattle] [14], leaf and root as a decoction against lices, and as a bath against scabs [17], root as a decoction and wash against lice and scabs [sheep] [9]
			*Juglans regia*	leaf against flies as a rub [29]	*Urginea maritima* (L.) Baker (Asparagaceae)	bulb in oil [16], or placed in shed as a repellent [cattle] [13]
			*Nicotiana tabacum*	leaf as a wash against lice, scabs and moths [20,33]	*Veratrum album*	aerial part as a tea [sheep] [16], root [calves] [55]
			*Persica vulgaris* Mill. (Rosaceae)	sap of the ground leaf smeared onto the body against flies [pig] [33]		
			*Polygonum lapathifolium*	leaf as a wash against lice, scabs and moths [26,30]		
			*Rumex crispus*	root against scab as a decoction [26]		
			*Tussilago farfara* L. (Asteraceae)	leaf against scab as a decoction [26]		
			*Veratrum album*	root dried and smeared onto the hair [19,20,22,25,33,41,48], or boiled with the leaf sap of *Brassica oleracea* against lice [sheep, cattle], or with goose fat [hen] [30]		
			cart-grease	as a rub against lice [34]		
			copper sulphate	in water with vinegar as a wash [sheep] [34]		
			fat	rancid fat against lice [hen], or with mercury [34]		
			lye-ashes	smeared onto the body [pig, sheep] [33]		
			petroleum	smeared onto the body [pig, sheep] [33]		
			potash-lye	in water as a bath [poultry] [33]		

*Number superscipts refere to the specific villages studied in Covasna: Biborţeni^1^, *Bodoş* ^2^ *,* Filia^3^, Racoşul de Sus^4^, Băţanii Mici^5^, Băţanii Mari^6^, Herculian^7^, Tălişoara^8^, Aita Seacă^9^, Ozunca-Băi^10^, Vârghiş^11^, Valea Zălanului^12^. Full botanical citations for plants documented in this study are provided in Table 2.

A total of 45 ingredients were documented in this survey, including 26 plant taxa (18 wild and 8 cultivated species; 57.8%), 2 animals (4.4%), and 17 animal-based substances, minerals or materials of other source (37.8%). Considering the frequency of citations, the use of *Allium sativum, Aristolochia clematitis, Armoracia rusticana, Potentilla anserina, Rumex acetosella,* and *R. obtusifolius,* as well as *Mustela nivalis,* “szénamurha”, powdered glass, sugar and water showed the highest prevalence (Tables 2 and 3).

The highest number of remedies involving plants were for the treatment of diarrhea (7 taxa), as anthelmintics (6), for rumination (5), stomach problems and wounds (4), while a few taxa were cited for mastitis (3), respiratory ailments and as a repellent drug (2) (Table 2). Local names of plants varied from 1 to 6 per species. Some names correspond with the official Hungarian terminology using in single form or with vernacular names together (vernacular names are listed in italics in Table 2).

Regarding the plant parts used, the whole herb was the most frequently used part of the cited taxa (21.9%) followed by leaf and fruit (18.6% each), root (15.6%), bark (9.4%), flower (6.3%), pseudofruit, seed and bulb (3.2% each) (Table 4). Herbal remedies were applied internally and externally as a single tea (40%) or tea mixture (2.8%), in raw form as fodder (37.2%), in washes (8.6%), rubbing agents (5.7%), creams and liniments (2.8% each). Plants containing toxic compounds (e.g. *Aristolochia clematitis*, *Veratrum album*) are only reported for external use. In the case of *Eryngium planum* and *Matricaria chamomilla,* two types of preparation were mentioned, similarly to the application of salt, vinegar and water.

Preparations based on other substances are commonly used with other materials (73.7%), with plants (5.3%), or in single form (21%) as a liniment, wash or fodder (Table 4). Although modern veterinary practice is expensive and not as easily available as homemade remedies, the use of injectable medicaments was also noted in the region (Table 3).

**Figure 3:**
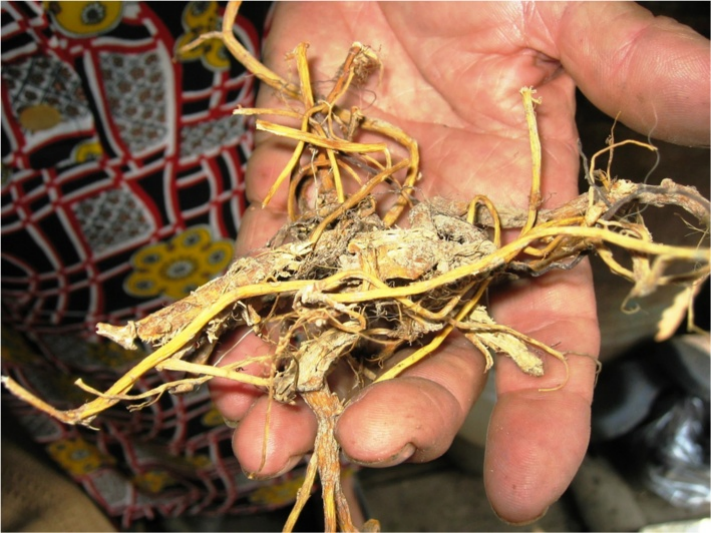
Root of *Gentiana asclepiadea* L.

Comparing data recorded in the selected 12 villages, the use of *Armoracia rusticana* for respiratory illness in horses, *Rumex* spp. for diarrhea, and the application of sugar and glass powders for cataracts in cattle proved to be consistent and a commonly used treatment in each community surveyed (Table 4). Intracultural variance was documented in the frequency of some records, such as in the use of *Allium sativum* as an anthelmintic drug (in 10 villages), *Aristolochia clematitis* for wounds and *Potentilla anserina* against diarrhea (in 9 villages). There were also some interesting cases of unique ethnoveterinary practices that were restricted to one village each. For example, *Gentiana asclepiadea* (Figure 3) was used with milk for stomach disorders; clay or water with salt for mastitis; *Juniperus communis,* “szénamurha” with urine and turpentine for respiratory ailments; cooking oil, *Cucurbita pepo* and *Petroselinum crispum* to improve rumination; the use of *Eryngium planum* and *Euphorbia amygdaloides* for wounds; *Quercus* species for diarrhea and as an anthelmintic drug used similarly to whey powder; “hótszén” against diarrhea; and *Petroselinum crispum* to dispel the placenta in cattle after delivery.

Some similarities were found between the indications reported earlier in other Transylvanian regions and other countries, and the present uses of home remedies for mastitis, skin problems, diarrhea, cataract, and in anthelmintic and diuretic drugs (Table 4).

**Figure 4:**
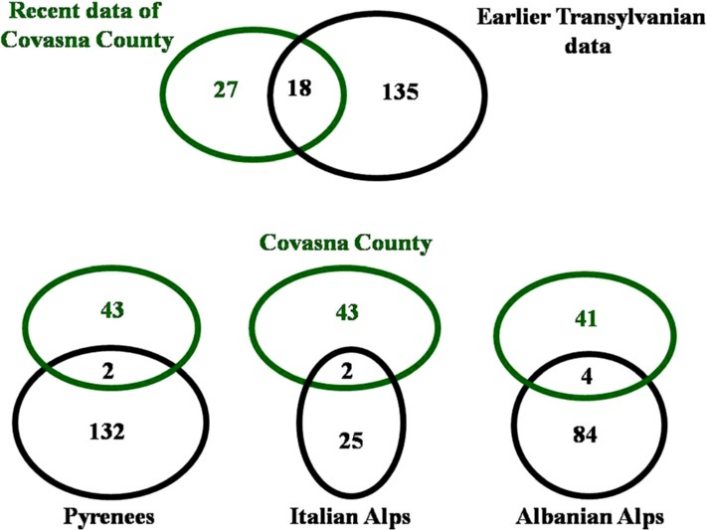
Overlaps of ethnoveterinary data involving plant- and animal-based materials and other substances used in Covasna County, Pyrenees, Italian and Albanian Alps [3,7,10,11,16,53-57,59-62].

Compared to the earlier records in Transylvania, we found 18 similar uses of the following (Table 4, Figure 4): *Calendula officinalis* flowers, as well as water with vinegar and salt to treat mastitis; *Armoracia rusticana* for respiratory ailments in Racu [34] and Lueta [47]; *Aristolochia clematitis* for external injuries in Ţara Călatei [21] and Homoród; and *Euphorbia amygdaloides* in Homoród [28,30] and Ghimeş [26]. *Allium sativum* has been documented in Romania for its widespread use as a vermifuge [29,34], similar to reports from Spain [3], Algeria [8], and Italy [14]. Similar to our findings, *Quercus* spp. fruits have been reported as vermifuges in Homoród [28]. The anti-diarrheal effect of *Q. rubra* has been observed in Morocco [8], and similar use of *Q. ilex* ssp. *ilex* have been reported in Catalonia [16], corresponding to our data on *Quercus petraea* and *Q. robur*. Furthermore, the use of *Artemisia absinthium* has been reported for diarrhea in Homoród [28], while for *Rumex* species (which are well-known for their anti-diarrheal effects), the use of *R. acetosella* has also been described in Lueta [47] and Croatia [5]. In contrast to the documented use of *Petroselinum crispum* leaves in the present work, the root has been recorded as a diuretic drug in Racu [34], Ţara Călatei [21], and Ghimeş [19,20].

For respiratory disorders, the cones of *Juniperus communis* were mentioned as a fodder in our survey, but as a tea in Moldova [25], while in Algeria the leaf of *J. phoenicea* has been documented [8]. The use of *Salix alba* has been similarly reported for rumination in Homoród [28,30], while *S. purpurea* has been documented in Albania [10]. In addition to the treatment of rumination disorders, *Petroselinum crispum* is used by itself in Covasna, but with bran in Trei Scaune [22]. In contrast to the use of *Polygonum minus*, the leaf of *P. lapathifolium* has been observed for wounds in Ghimeş [26]. The seed of *Cucurbita pepo* has been used for skin problems by itself in Covasna, but with castor oil in Harghita County [29].

*Veratrum album* root has been reported as a widespread repellent and antiparasitic drug in Covasna [41], Ghimeş [19], Ţara Călatei [21,48], Trei Scaune [22], Moldova [25,33], and Lueta [47]. *Juglans regia* leaves have also been reported for their use as a repellent in Harghita County [29]. Similar use of sugar and powdered glass has been observed for cataract in Ghimeş [19], Ţrei Scaune [22], and Moldova [33].

Comparison is represented between our data and those of the mountain regions of Pyrenees, Italian and Albanian Alps, which covers the overlap of each ingredient (Figure 4). Similar to our records, the use of *Achillea millefolium* was mentioned for rumination and digestive disorders in the Lombardy [54] and Albanian Alps [56]. As anti-lice treatment, the root of *Veratrum album* was mentioned in Italy [55], and the aerial part of the plant in Catalonia [16]. Similar to our records, *Allium sativum* was documented as vermifuge in Galicia [3], *Rumex* species against diarrhea [64], and haemostatic use of cobweb and milk for intestinal aches in the Albanian Alps [7].

Rituals and beliefs connected to local uses were sporadically mentioned in the region. The skin of *Mustela nivalis* was reported against udder inflammation caused by weasel bites, similarly to data recorded in Uz-valley [49]. To stimulate urination, animals should hear the sound of rippling stream or poured water (Table 4).

Some of the present uses were not found in earlier Transylvanian reports nor in databases of other countries, such as remedies for “hotness” and for applications following delivery (e.g. for stimulating expulsion of the placenta). In addition, several practices are no longer used today, but rather survive only in the memory of the villagers, such as the use of cobweb for wounds, “hótszén” for diarrhea, and the placement of lice into the urethra as a diuretic.

## Conclusions

From an ethnoveterinary point of view, Covasna has proven to be one of the most interesting regions of Romania due to the diversity of knowledge concerning plant-, animal- and other substances-derived remedies. These traditions are practiced mostly by the more elderly subset of the population, forming a significant part of the local animal healthcare and cultural heritage of the region. Although some data survive only as memories from the past, people are proud of their traditional knowledge, which is still maintained in rural areas. In addition, holders of this knowledge have an important role as natural resource managers.

Although ethnoveterinary service is cheaper and easily available compared to modern veterinary medicine and pharmaceuticals, factors such as the size and prevalence of herds, as well as the frequency of citation of traditional ethnoveterinary practices are diminished in comparison to earlier records of Romania, and other European countries. This change has also been influenced by shifting socio-cultural factors concerning local economies and emigration patterns, as well as less frequent opportunities for the vertical transmission of traditional knowledge. Future studies to support our further understanding of the role that ethnoveterinary practices can play in managing animal health are certainly merited. Such studies are useful not only for the purposes of folkloric preservation, but can also form a foundation on which to support sustainable development efforts aimed at promoting environmentally friendly, cost-effective means of maintaining livestock health.
